# Bibliometric analysis of preoperative radiotherapy for locally advanced rectal cancer: evolution and future

**DOI:** 10.3389/fmed.2025.1518640

**Published:** 2025-02-17

**Authors:** Zhen-Hong Weng, Hao-Kai Hu, Zi-Shan Zhou, Li-Sheng Huang, Bin-Bin Chen, Jia-Rui Lin

**Affiliations:** ^1^Department of Gastrointestinal Surgery, Cancer Hospital of Shantou University Medical College, Shantou, Guangdong, China; ^2^Department of Health Management Clinic, Cancer Hospital of Shantou University Medical College, Shantou, Guangdong, China; ^3^Department of Radiotherapy, Cancer Hospital of Shantou University Medical College, Shantou, Guangdong, China

**Keywords:** bibliometrics, preoperative, radiotherapy, locally advanced, rectal cancer

## Abstract

**Background:**

Preoperative radiotherapy of rectal cancer has been a hot topic of research in recent years with the introduction of total neoadjuvant therapies and immunotherapeutic agents. We utilized bibliometrics and visualization analysis to examine studies in this field, aiming to identify current hotspots and research trends.

**Method:**

We searched the Web of Science database for all publications related to preoperative radiotherapy of rectal cancer in the past 10 years. Using bibliometric analysis software, such as VOSviewer, CiteSpace and R-studio, we extracted and analyzed the data, summarizing the publication output of countries, institutions, authors, and journals in this field, and analyzing their relationships. We also summarized the keywords, burst words, and most cited articles, and analyzed the relationships among them.

**Results:**

We found 794 publications in the field, sourced from 217 journals or books, involving 5,805 authors from various organizations and countries. Through bibliometric analysis, we observed a growing trend in the number of publications in preoperative radiotherapy of rectal cancer over the past 10 years. China, United States and Italy were the top countries in terms of publication output. Sun Yat-sen University, Fujian University, and Fudan University were the top three medical centers in terms of publication output, while Leiden University from Netherlands led globally in terms of citation impact. Professor Zhen Zhang, Sanjun Cai, and Ji Zhu were the top three authors with the highest publication output. The most highly cited journals in this field includes “The Lancet Oncology,” “J Clinical Oncology,” and “Annals of Oncology.” Journals such as “Radiotherapy and Oncology,” “Frontiers in Oncology,” and “BMC Cancer” have the highest number of articles published. Based on the analysis of keywords and burst words, we found that “preoperative chemoradiation” and “oral capecitabine” were the research hotspots before 2016, while the focus shifted to “short-course radiotherapy” and “long-term outcomes” after 2017. Currently, the most frequently cited publications mainly summarize multicenter clinical studies and total neoadjuvant treatment models and immunotherapy.

**Conclusion:**

Research on preoperative radiotherapy of rectal cancer is increasing year by year, and attracting attention from high-cited journals such as “The Lancet Oncology,” “JCO,” and “Annals of Oncology.” Based on current data, the total neoadjuvant treatment models and radiation combined with immunotherapy are the research trends.

## 1 Introduction

According to recent statistics, colorectal cancer ranks among the top three most common cancers globally and is the second leading cause of cancer-related deaths (1). In China, colorectal cancer has shown a notable increase in incidence in recent years, distinguishing it as a malignancy with one of the fastest increases in incidence (2). Unlike western countries, rectal cancer comprises a significantly high proportion of colorectal cancers in China, accounting for 60% of cases, predominantly occurring at the mid to low rectum (3). Treatment for this type of cancer is compromised due to pelvic space constraints and the imperative of sphincter preservation, making treatment strategies more complex compared to colon cancer (4).

Current international guidelines uniformly advocate for neoadjuvant radiotherapy for locally advanced mid to low rectal cancer to reduce local recurrence rates (5). However, previous studies have indicated that the addition of radiotherapy does not necessarily enhance long-term survival (6). Recent years have witnessed substantial changes in the approach to neoadjuvant therapy for rectal cancer, with the introduction of total neoadjuvant therapies and immunotherapeutic agents. Globally, discussions surrounding this topic have intensified, accompanied by a continuous influx of relevant research (7–10). Consequently, a systematic review of studies on neoadjuvant radiotherapy for rectal cancer and an analysis of emerging treatment modalities will provide crucial insights for researchers planning studies in this field.

Bibliometrics is widely utilized for quantitative assessment of literature and exploration of research trends (11, 12). Tools such as VOSviewer, CiteSpace, and R-studio currently predominate for visualizing and analyzing scientific paper trends and patterns. They help in understanding the relationships among countries, research institutions, and journals within a research field, as well as in identifying and monitoring research hotspots (13–16). In this study, we employ these tools to analyze literature on neoadjuvant radiotherapy for rectal cancer over the past decade, aiming to elucidate the evolution of research trends and hotspots. To our knowledge, there is currently a lack of comprehensive bibliometric analysis evaluating clinical research on neoadjuvant radiotherapy for rectal cancer over the last decade.

The objective of this study is to use bibliometric methods to analyze the network relationships in literature related to neoadjuvant radiotherapy for rectal cancer, visually presenting data to demonstrate the relationships among different institutions, authors, journals, and countries involved in these studies, and to explore research hotspots and trends.

## 2 Methods

### 2.1 Search strategy

A literature search was conducted using the Web of Science database. The search strategy we used is as follows: MeSH: TS = (“Rectal Neoplasm” OR “Rectum Neoplasm” OR “Rectal Tumor” OR “Rectal Cancer” OR “Rectum Cancer”) AND TS = (“Radiotherapy” OR “Radiation Therapy” OR “Radiation Treatment”).

### 2.2 Inclusion and exclusion criteria

Inclusion criteria encompassed: (1) clinical research articles pertaining to neoadjuvant radiotherapy for rectal cancer; and (2) reviews, meta-analyses, clinical trial designs, guidelines, and conference abstracts related to neoadjuvant radiotherapy for rectal cancer.

Exclusion criteria included book chapters, animal studies, and cadaver investigations.

### 2.3 Data extraction and visualization methods

Data extraction: all information was exported from the Web of Science in text format, including author names, article types, citations, countries, digital object identifiers (DOIs), impact factors, journals, institutions, keywords, sample sizes, study types, titles, and publication years.

Data analysis: data were analyzed using VOSviewer, CiteSpace and R-studio for co-citation and visualization analysis (17–19). CiteSpace constructs citation networks based on document relationships, employing algorithms to analyze network structures and characteristics to identify research hotspots and key literature (14). The main steps were as follows: (1) importing the dataset into CiteSpace, (2) adjusting the years per slice (1 year), (3) selecting node types (i.e., cited journal or keywords) and the algorithm for link strength (cosine), and (4) setting the selection criteria (the top 20 most cited or frequently occurring items in each slice). VOSviewer utilizes clustering and visualization techniques to transform literature data into multidimensional networks, revealing scientific collaboration networks and thematic evolution trends using density and layout algorithms (15). VOSviewer employs Multidimensional Scaling (MDS) and clustering algorithms (such as those based on adjacency matrices) to cluster documents and uncover relationships between different topics or research areas. These methods rely on statistical clustering techniques, particularly K-means clustering and hierarchical clustering. The main steps were as follows: (1) importing the dataset into VOSviewer, (2) selecting the type of analysis (i.e., co-authorship) and the unit of analysis (i.e., countries, organizations, or authors), (3) selecting the counting method (full counting), and (4) setting analysis parameters (default values).R provides extensive literature analysis packages and user-friendly syntax for efficient processing of large-scale literature data, extracting valuable insights to uncover developmental trends and critical influencing factors in academic fields. The main steps were as follows: (1) importing the dataset into R software, (2) selecting the type of analysis (i.e., “co-occurrence network,” “thematic evolution,” and “main information”), and (3) setting analysis parameters. The complementary strengths of these tools ensure comprehensive analytical outcomes. Finally, results were imported into WPS software for table preparation.

## 3 Results

From 2014 to 2024, 794 publications were retrieved in the field of study, originating from 217 journals or books, involving 5,805 authors from various organizations and countries. These articles collectively cited 12,403 references from journal publications. Among them, several articles were cited more than 20 times.

### 3.1 Trends in research

**Figure 1:**
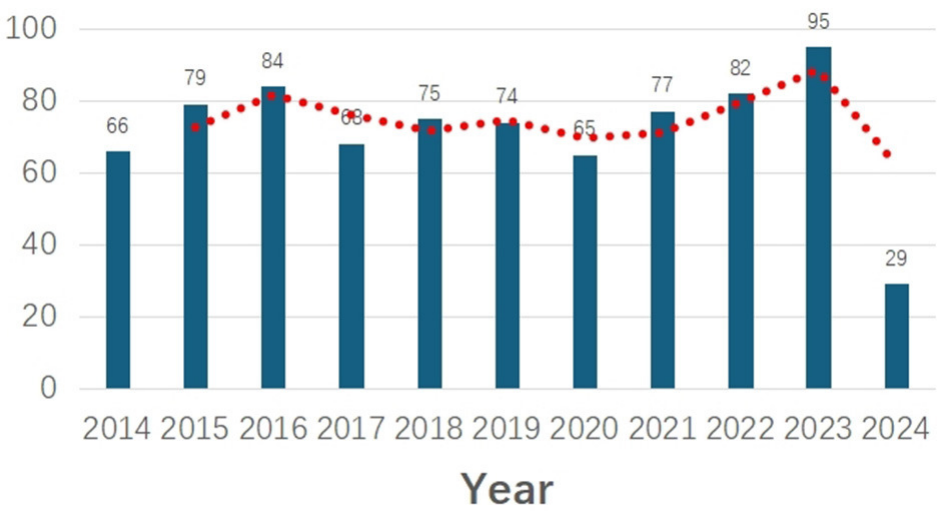
Annual scientific production.

Figure 1 depicts the trends in publications related to preoperative radiotherapy for rectal cancer, showing fluctuations in annual publication counts over the past decade. Two peaks are notable in 2016 and 2023, with a gradual increase observed since 2020. The highest annual growth rates were recorded in 2014 and 2023.

### 3.2 Co-citation analysis

Using VOSviewer, a collaboration network map was generated, where nodes represent countries/regions, institutions, and authors. Larger nodes indicate higher productivity or more frequent citations. Connections between nodes represent co-occurrence or co-citation relationships, with thicker lines indicating stronger associations (literature). Different colors distinguish publications from different years: blue represents pre-2018, green represents around 2019, and yellow represents post-2020 publications.

#### 3.2.1 Bibliometric analysis of countries and institutions

**Figure 2:**
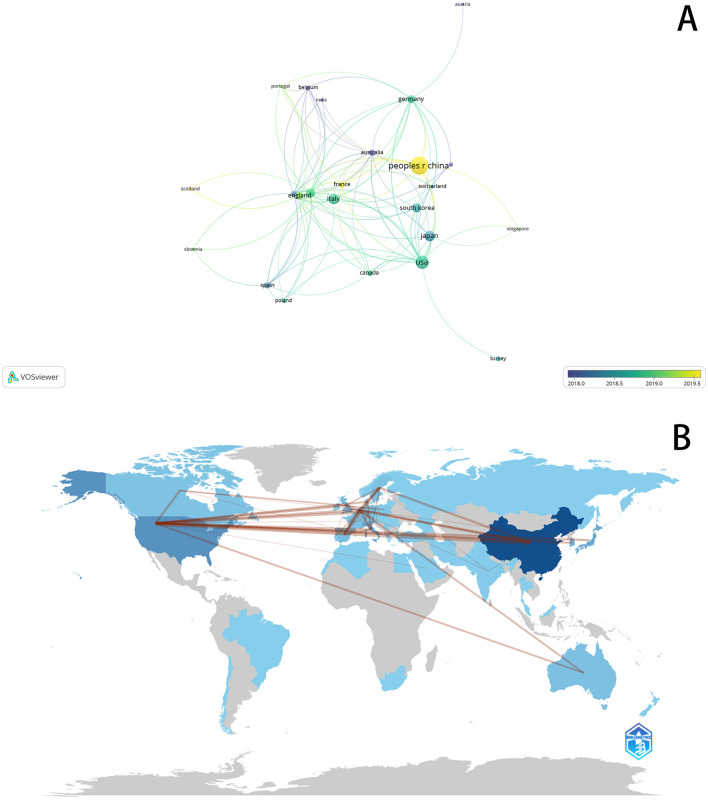
**(A)** Visualizes the collaborative connection between countries. **(B)** Country Collaboration map.

The top five countries by publication volume were China (241 papers), the United States (116 papers), Italy (73 papers), Japan (70 papers), and South Korea (54 papers; Table 1). Before 2018, Japanese and South Korean publications dominated. Around 2019, publications from the United States and Europe became prominent, while post-2020 contributions predominantly came from China (Figure 2A). A world map illustrates each country's contributions to the field (Figure 2B), using color intensity to denote publication volume and line density to depict collaboration between countries. Notably, China, the United States, and several European countries exhibited close collaborative ties, with China and the United States showing the strongest connections.

**Table 1:** Summarizes the top 10 countries/Regions with the highest volume of publications.

**Rank**	**Country/ region**	**Publication**	**Total citations**	**Average citation per paper**
1	China	214	3,233	15
2	USA	116	3,156	27
3	Italy	73	1,184	16
4	Japan	70	1,291	18
5	South Korea	54	780	14
6	Netherlands	52	2,251	43
7	England	38	733	19
8	Germany	37	1,195	32
9	Spain	27	1,169	43
10	Taiwan	27	217	8

**Figure 3:**
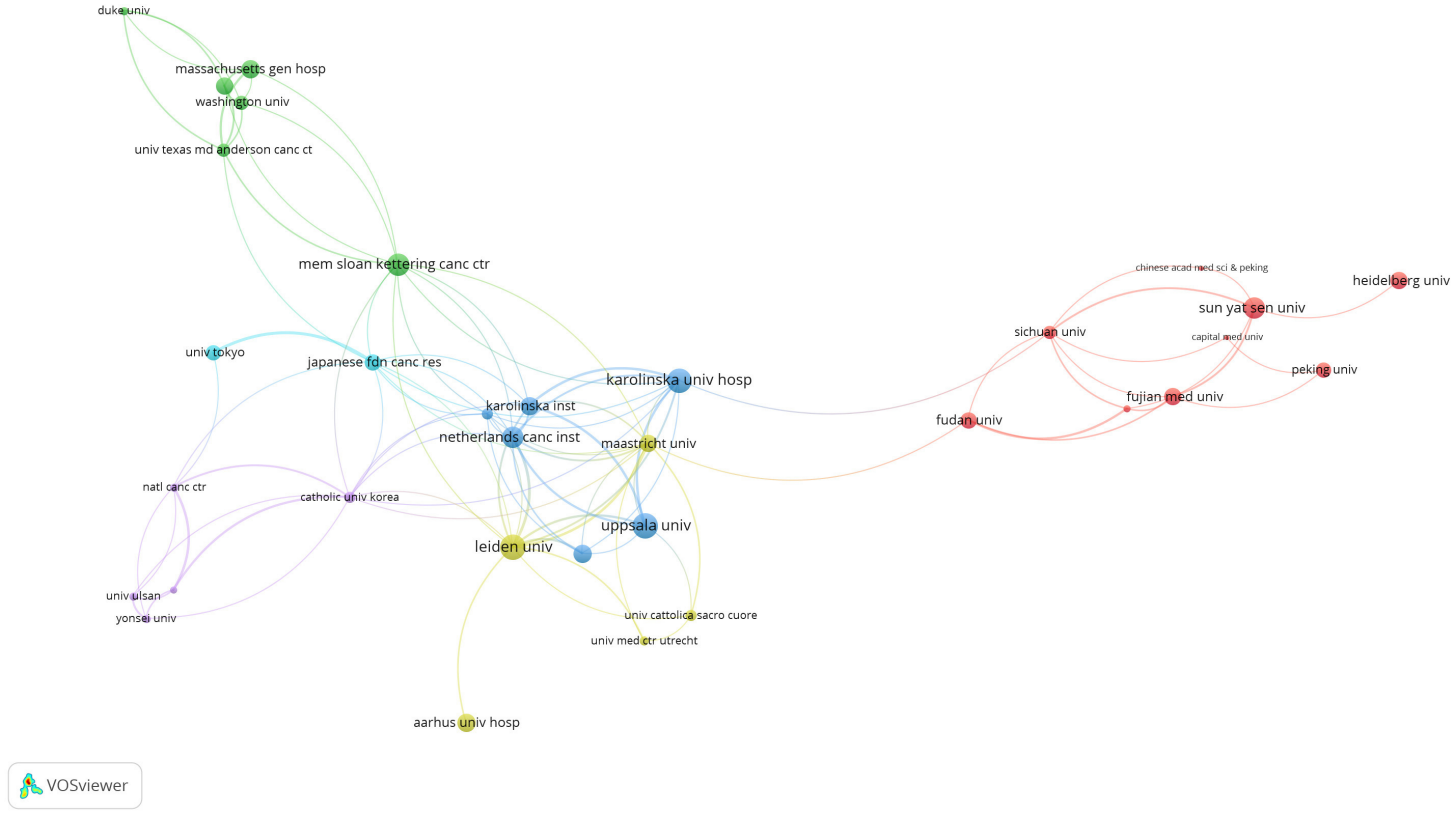
Visualizes the citation relationships between research institutions.

The top five institutions by publication volume were Sun Yat-sen University, Fujian University, Fudan University, Sichuan University, and Leiden University (Table 2). Sun Yat-sen University led with 37 papers, largely due to multiple affiliated hospitals contributing to research in this area (6, 20–25). Notably, the Sixth Affiliated Hospital of Sun Yat-sen University, led by Professor Jianping Wang in 2019, published the FORWARD study in JCO, which became one of the top ten most cited papers in the field (6). Among the top five universities by publication volume, Fujian University stood out for its strong collaborative relationships with the other four institutions, particularly bolstered by Professor Pan Chi's involvement in several prospective randomized controlled studies. Despite four of the top five institutions being Chinese, based on publication volume, only two Chinese institutions ranked in the global top ten for citations: Sun Yat-sen University (6th) and Fujian University (10th). Leiden University, ranked 5th by publication volume and based in the Netherlands, led globally in terms of citation impact (Figure 3).

**Table 2:** Summarizes the top 10 research institutions in terms of publication volume.

**Rank**	**Institution**	**Country**	**Document**	**Total citations**	**Average citation per paper**
1	Sun Yat Sen univ	China	37	1,027	28
2	Fujian med univ	China	26	661	26
3	Fudan univ	China	26	586	23
4	Sichuan univ	China	20	376	19
5	Leiden univ	Nertherland	20	1,448	72
6	Univ Texas md Anderson Caner ctr	USA	19	427	22
7	Peking univ	China	18	535	30
8	Univ Tokyo	Japan	17	510	30
9	Yonsei univ	South Korea	14	169	12
10	Japanese fdn canc res	Japan	12	609	51

#### 3.2.2 Bibliometric analysis of authors

Author research contributions were evaluated based on the number and quality of their publications. A total of 5,805 authors contributed to the 794 articles in this field. Table 3 lists the top 10 authors by publication volume, along with their total citations and average citations per article. The top three authors were all from Fudan University Affiliated Hospitals in China: Professor Zhen Zhang (17 papers), Dr. Sanjun Cai (14 papers), and Professor Ji Zhu (13 papers). Professor Zhang is the Director of Radiation Oncology at Fudan University Affiliated Cancer Hospital, while Dr. Cai serves as the Director of Gastrointestinal Surgery. Ranking 4th and 5th by publication volume are Dr. Yong Cai from Peking University Cancer Hospital and Dr. Lin Wang from Gastrointestinal Surgery. The top two authors by citation count were Professor Bengt Glimelius from Uppsala University in Sweden and Dr. Corrie Marijnen from the Netherlands Cancer Institute. Authors Zhang Zhen, Wang Lin, and Cai Sanjun from China ranked 3rd to 5th in terms of citations.

**Table 3:** Summarizes the 10 authors with the highest publication volume.

**Rank**	**Author**	**Publication**	**Total citations**	**Average citation per paper**	**H-index**
1	Zhang Zhen	17	445	26	11
2	Cai Sanjun	14	352	25	11
3	Zhu Ji	13	317	24	9
4	Cai Yong	11	74	7	6
5	Wang lin	11	440	40	11
6	Kawai Kazushige	10	162	16	6
7	Marijnern Corrie a.m.	10	1,172	117	9
8	Chi pan	10	62	6	7
9	Ishihara Soichiro	9	193	21	7
10	Glimelius Bengt	9	1,320	147	8

**Figure 4:**
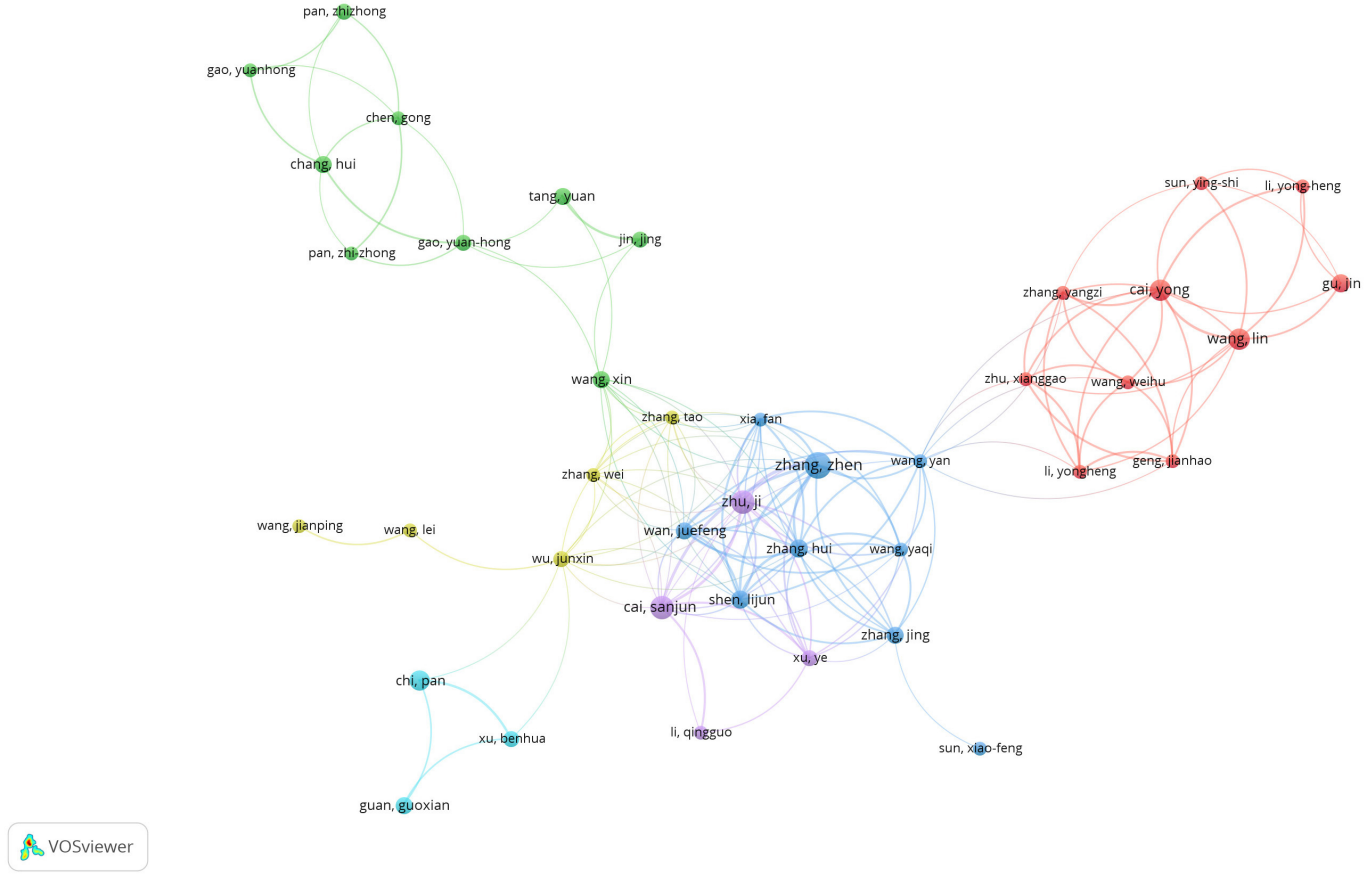
Visualizes the collaborative connection between research authors.

Collaboration among researchers integrates resources and enhances research quality. Analyzing author relationships allows researchers to identify existing collaborations and explore potential joint projects. Therefore, we utilized VOSviewer to construct a collaboration network map of authors (Figure 4), revealing approximately six clusters. Chinese authors were led by Zhang Zhen, Zhu ji, Wang Lin, Zhang tao, Wang xin, and Pan Chi, respectively and contributed the majority of the research. However, Chinese researchers have fewer collaborative relationships with researchers from Europe and Japan.

#### 3.2.3 Bibliometric analysis of journals

**Figure 5:**
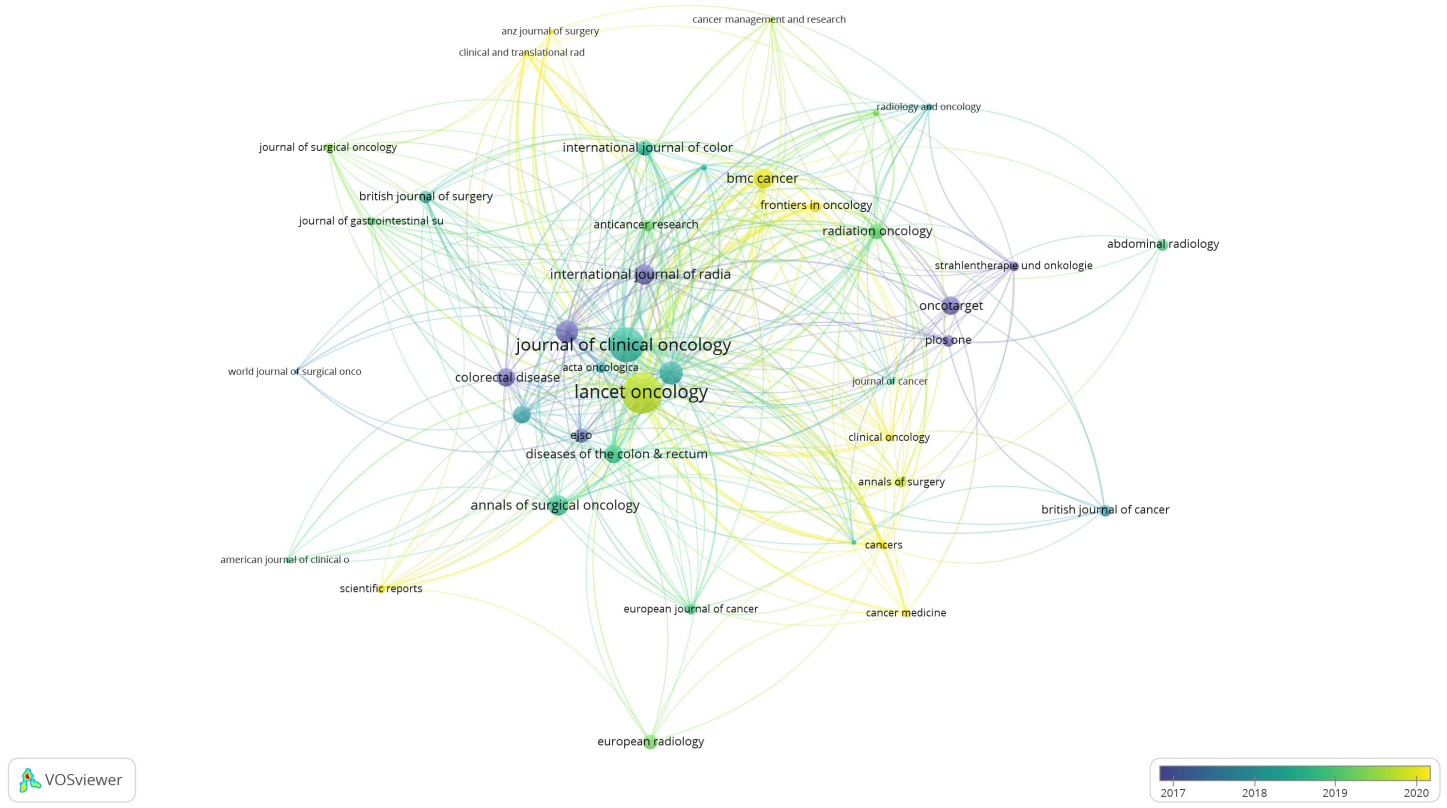
Visualizes the citation relationships between journals.

Table 4 summarizes the 10 most highly cited journals in this field, along with the number of articles they have published: The Lancet Oncology (6 articles, cited 2,154 times, averaging 359 citations per article); J Clinical Oncology (7 articles, cited 1,538 times, averaging 220 citations per article); Radiation and Oncology (31 articles, cited 682 times, averaging 22 citations per article); Annals of Oncology (8 articles, cited 651 times); Ann Surgery Oncology (8 articles, cited 550 times); Int J Radiation Oncology (17 articles, cited 523 times); BMC Cancer (26 articles, cited 519 times); Clinical Cancer Research (4 articles, cited 505 times); Oncotarget (21 articles, cited 460 times); and Dis Colon Rectum (23 articles, cited 448 times). Table 5 summarizes the top 10 journals by publication volume. The highest was “Radiotherapy and Oncology,” followed by “Frontiers in Oncology,” “BMC Cancer,” “Radiation Oncology,” and “Diseases of the Colon and Rectum.” Figure 5 illustrates citation relationships among the journals with the highest citations. Node colors indicate that highly cited articles in Annals of Oncology mostly predated 2018, while those in JCO were primarily from 2018 to 2019; and recent highly cited articles were mainly from The Lancet Oncology and BMC Cancer.

**Table 4:** Summarizes the top 10 journals with the highest citations.

**Rank**	**Journal**	**Country**	**Publication**	**Total Citations**	**Average citation per paper**	**CR**	**IF (5 years)**
1	Lancet oncology	USA	6	2,154	359	Q1	42.2
2	Journal of clinical oncology	USA	7	1,538	218	Q1	37.4
3	Radiotherapy and oncology	Netherland	31	682	22	Q1	5.4
4	Annals of oncology	England	8	651	81	Q1	38.2
5	Annals of surgical oncology	USA	8	550	69	Q2	4
6	International journal of radiation oncology biology physics	USA	17	523	31	Q1	6.1
7	BMC cancer	England	26	519	20	Q2	3.8
8	Clinical cancer research	USA	4	505	126	Q1	11.1
9	Oncotarget	USA	21	460	22	Q2	5.312
10	Diseases of the colon and rectum	USA	23	448	19	Q2	4

**Table 5:** Summarizes the top 10 journals with the highest publication volume.

**Rank**	**Journal**	**Country**	**Publication**	**Total citations**	**CR**	**IF (5 years)**
1	Radiotherapy and oncology	Netherlands	31	682	Q1	5.7
2	Frontiers in oncology	Switzerland	28	172	Q2	5.0
3	BMC cancer	England	26	519	Q2	3.9
4	Radiation oncology	England	25	323	Q1	3.5
5	Diseases of the colon and rectum	USA	23	448	Q1	3.3
6	Anticancer research	Greece	23	178	Q4	2.1
7	Clinical colorectal cancer	USA	19	380	Q2	3.7
8	International journal of colorectal disease	Germany	18	333	Q1	2.6
9	International journal of radiation oncology biology	USA	17	523	Q1	6.9
10	Cancers	Switzerland	16	99	Q1	5.8

Impact Factor (IF), originally introduced by Eugene Garfield, is often used as a measure of journal quality, representing a 2-year rolling average of citations (26, 27). The top five journals by citation count have IFs of 42.2, 37.3, 5.4, 38.2, and 4. The top five journals by publication volume have IFs of 5.7, 5.0, 3.9, 3.5, and 3.3 (Tables 5, 6).

**Table 6:** Summarizes the top 10 publications with the highest citations.

**Rank**	**Title**	**Journal**	**First author**	**Year**	**Local Citations**
1	Short-course radiotherapy followed by chemotherapy before total mesorectal excision (TME) versus preoperative chemoradiotherapy, TME, and optional adjuvant chemotherapy in locally advanced rectal cancer (RAPIDO): a randomized, open-label, phase 3 trial	The lancet oncology	Renu R. Bahadoer	2020	120
2	Oxaliplatin added to fl uorouracil-based preoperative chemoradiotherapy and postoperative chemotherapy of locally advanced rectal cancer (the German CAO/ARO/AIO-04 study): fi nal results of the multicentre, open-label, randomized, phase 3 trial	The lancet oncology	Claus Rödel	2015	97
3	Neoadjuvant chemotherapy with FOLFIRINOX and preoperative chemoradiotherapy for patients with locally advanced rectal cancer (UNICANCER-PRODIGE 23): a multicentre, randomized, open-label, phase 3 trial	The lancet oncology	Thierry Conroy	2021	79
4	Capecitabine and oxaliplatin in the preoperative multimodality treatment of rectal cancer: surgical end points from national surgical adjuvant breast and bowel project trial R-04	Journal of clinical oncology	Michael J. O'Connell	2014	66
5	Optimal fractionation of preoperative radiotherapy and timing to surgery for rectal cancer (Stockholm III): a multicentre, randomized, non-blinded, phase 3, non-inferiority trial	The lancet oncology		2017	56
6	Long-course oxaliplatin based preoperative chemoradiation vs. 5 × 5 Gy and consolidation chemotherapy for cT4 or fixed cT3 rectal cancer: results of a randomized phase III study	Annals of oncology	K. Bujko	2016	50
7	Organ preservation in patients with rectal adenocarcinoma treated with total neoadjuvant therapy	Journal of clinical oncology	Julio Garcia-Aguilar	2022	40
8	Effect of Interval (7 or 11 weeks) between neoadjuvant radiochemotherapy and surgery on complete pathologic response in rectal cancer: a multicenter, randomized, controlled trial (GRECCAR-6)	Journal of clinical oncology	J'er'emie H	2016	38
9	Modified FOLFOX6 with or without radiation versus fluorouracil and leucovorin with radiation in neoadjuvant treatment of locally advanced rectal cancer: initial results of the Chinese FOWARC multicenter, open-label, randomized three-arm phase III trial	Journal of clinical oncology	Yanhong Deng	2016	37
10	Long-course preoperative chemoradiation vs. 5 x 5 Gy and consolidation chemotherapy for clinical T4 and fixed clinical T3 rectal cancer: Long-term results of the randomized Polish II study	Annals of oncology	B. Ciseł	2019	31

**Figure 6:**
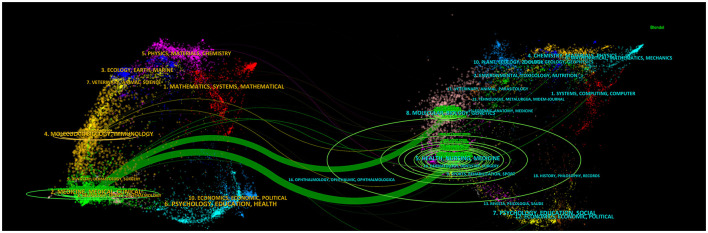
The journal dual-map produced through CiteSpace.

Figure 6 depicts an overlay of journal dual maps. The left side shows citing journal clusters, with different colors representing various thematic clusters at the forefront of current research in the field. Specific journals included “Radiotherapy and Oncology.” The right side shows cited foundational literature, originating mainly from publications in health, nursing, medicine, molecular biology, and genetics, including “The Lancet Oncology,” “New England Journal of Medicine,” and “J Clinical Oncology.”

### 3.3 Statistical analysis of keywords

**Figure 7:**
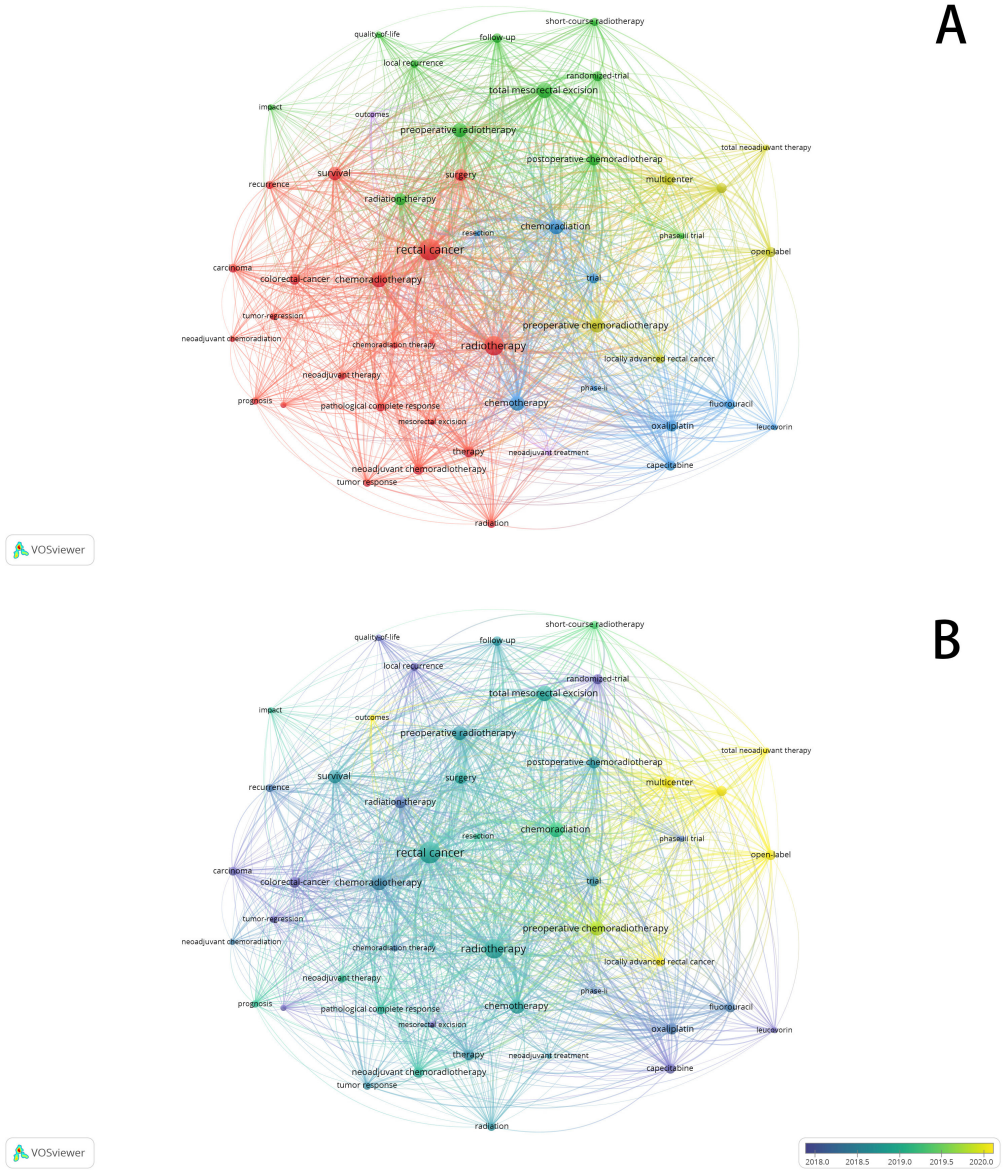
**(A)** Visualizes the co-occurrence analysis of keywords (cluster view). **(B)** Visualizes the co-occurrence analysis of keywords (timeline view).

Keywords are words or phrases used to describe the main theme, content, and focus of an article. They are intended to aid readers in understanding the subject matter of the literature. Consequently, high-frequency keywords often reflect current issues within a research field. Analyzing keywords over different time periods also provides insights into changes in research emphasis within that field. We employed VOSviewer and CiteSpace to analyze and depict the current status and hotspots of preoperative radiotherapy of rectal cancer research, presenting co-occurrence maps in two forms: cluster view and timeline view. In the visualization of keyword networks, larger node sizes indicate higher frequencies of co-occurrence for those keywords. Additionally, the thickness of lines between nodes represents the strength of the co-occurrence, with thicker lines indicating stronger relationships between nodes. Thus, larger nodes correspond to more significant keywords within the research domain. Using Lotka's law, this study identified keywords appearing 30 times or more (Figures 7A, B). “Rectal cancer” emerges as the largest node, followed by “chemoradiotherapy,” “total mesorectal excision,” “preoperative,” and “survival,” indicating current hotspots in preoperative chemoradiotherapy, surgical quality control, and prognosis research in this field. The timeline view also reveals varying keywords across different years. Before 2016, the focus was primarily on the selection of combined chemotherapy drugs during radiotherapy, ranging from 5-FU and capecitabine to oxaliplatin. From 2017 to 2019, research shifted toward surgical quality control and local recurrence rates. Post-2020, emphasis moved to long-term efficacy, comprehensive neoadjuvant therapies, multicenter clinical studies of advanced local rectal cancer, and immunotherapy. This shift underscores ongoing interest in treatment modalities and outcomes of chemoradiotherapy. Notably, keywords related to immunotherapy began appearing after 2020, suggesting potential future research trends in combined immunotherapy, comprehensive neoadjuvant therapy, and late-stage local rectal cancer.

### 3.4 Bibliometric analysis of burst keywords

**Figure 8:**
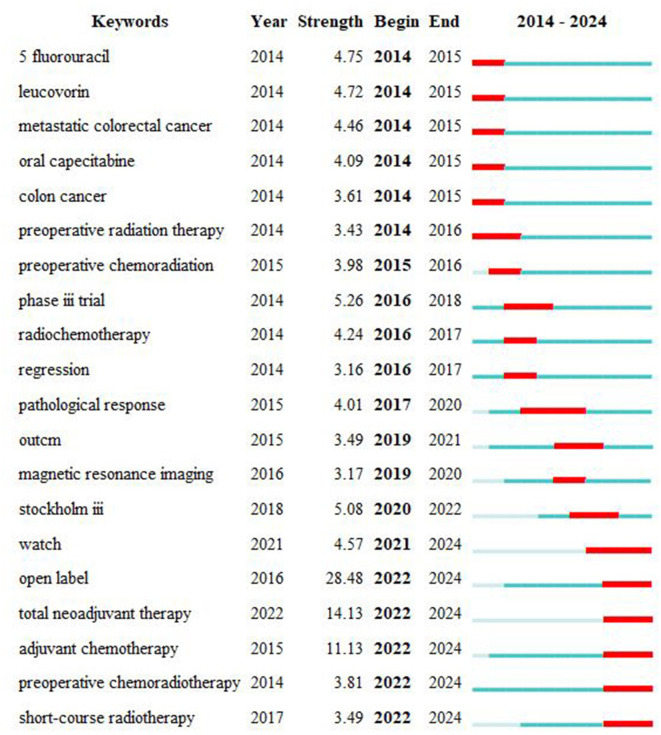
Top 20 keywords with the strongest citation bursts.

To better understand the developmental trajectory and future research hotspots in this field, CiteSpace was employed for burst analysis. The results (Figure 8) indicate bursts mainly occurring during 2014–2016 and 2020–2022. The first phase's burst keywords were primarily “preoperative chemoradiation,” “5 fluorouracil,” “oral capecitabine,” and “leucovorin,” highlighting a focus on preoperative chemoradiotherapy and concurrent chemotherapy drugs at that time. In the second phase, burst keywords included “adjuvant chemotherapy,” “total neoadjuvant therapy,” and “short-course radiotherapy,” indicating current industry focus on clinical research, comprehensive neoadjuvant modes, and short-term radiotherapy. Keywords with bursts exceeding an intensity of five included “open label,” “Stockholm III,” and “phase III trial,” reflecting a strong interest in multicenter prospective randomized controlled trials within this field. Improving current treatment modalities, including TNT mode, short-course radiotherapy, combined immunotherapy, and postoperative adjuvant therapy, remains pivotal research areas.

### 3.5 Article analysis

#### 3.5.1 Articles of the top 10 most cited

In this section, we summarize the top 10 most cited articles presented in Table 6 (6–8, 28–34).

#### 3.5.2 Articles on radiotherapy combined with immunotherapy

The reporting on radiotherapy combined with immunotherapy began in 2021 and consists predominantly of prospective single-arm studies, with ongoing trials mostly being prospective randomized controlled studies. We selected and analyzed two published studies for this review.

The first study, NRG-GI002 from the United States (9), published in 2021 in JAMA Oncology (IF 22.5), enrolled patients with locally advanced T3/T4/N2 rectal cancer, and employed the TNT regimen, involving 4 months of FOLFOX chemotherapy followed by random assignment into study and control arms. The study arm received long-course radiotherapy concurrently with up to six cycles of pembrolizumab, whereas the control arm received long-course radiotherapy alone. A total of 185 patients were enrolled. The primary endpoint, the neoadjuvant rectal (NAR) score, was 11.53 in the study arm compared to 14.08 in the control arm (*p* = 0.26). While the study arm appeared to show a decrease in NAR score, the difference between the groups was not statistically significant. The study also indicated no significant difference in treatment-related adverse events between the two groups, suggesting the addition of immunotherapy was safe.

The second study, UNION from China, published in 2024 in the Annals of Oncology (IF 56.7), was a randomized phase III trial (10). Patients with T3-4/N+ rectal adenocarcinoma were randomly assigned (1:1) to receive either short-course radiotherapy (SCRT) followed by two cycles of camrelizumab and CAPOX or long-course 60 Gy chemoradiotherapy (LCRT) followed by two cycles of CAPOX alone, respectively. A total of 232 patients were enrolled. The primary endpoint, pathological complete response (pCR) rate, was significantly higher in the SCRT plus immunotherapy group at 39.8% compared to 15.2% in the LCRT group (*p* < 0.001). There was no significant difference in the incidence of adverse events between the two groups. This study suggests that sequential short-course radiotherapy followed by immunotherapy can significantly improve short-term efficacy, with long-term outcomes still under follow-up.

## 4 Discussion

We conducted a systematic review using the Web of Science database to identify relevant literature on preoperative radiotherapy for rectal cancer published over the past decade, yielding a total of 794 articles. Our study reveals a global increase in publications in this field over the past decade, with a notable surge observed since 2023. Employing bibliometric analysis, we examined the developmental trends and future directions of this field. Our analysis explored the publication patterns across countries, institutions, authors, and journals, providing valuable insights that could assist scholars planning research in this domain.

### 4.1 Countries

A total of 55 countries worldwide have published papers in this field, with China, the United States, and Italy being the most prolific contributors. This reflects that radiotherapy for rectal cancer has become a globally recognized treatment modality, incorporated into major international guidelines. Analyzing highly cited articles from different countries in this field reveals distinct focuses: research from the United States predominantly emphasizes long-course radiotherapy and total neoadjuvant therapy (TNT), while significant European studies concentrate on the efficacy and safety of short-course radiotherapy. China, as both a high-incidence country for rectal cancer and the largest producer of publications, explores various strategies, including long-course, short-course radiotherapy, and TNT, with an emphasis on precise patient selection for different therapeutic approaches. However, despite the volume of publications, Chinese literature averages the lowest citation rates, indicating a need for future research to prioritize high-quality clinical studies. Temporal analysis shows that American and Japanese-Korean studies were primarily conducted before 2018, Italian studies predominated in 2018–2019, and Chinese research has surged mainly after 2020. This suggests that as economic development and research investment increase, China may emerge as a significant leader in future research in this field. A bibliographic coupling analysis reveals two distinct clusters in the international literature network: one primarily comprising China and the United States, and another consisting mainly of European countries such as Italy, the Netherlands, Sweden, and the United Kingdom. China maintains close research ties with the United States but less so with Japan, South Korea, and Singapore, potentially fostering broader research collaborations with Asian countries in the future.

### 4.2 Institutions and authors

This study identifies six distinct clusters of research institutions in this field, with the Chinese cluster operating relatively independently from the other five groups. Sun Yat-sen University emerges as the leading contributor within Chinese institutions and globally in terms of publication volume, yet it maintains limited connections with institutions outside China. Fudan University and Sichuan University have established links with European institutions, such as Maastricht University and the Karolinska Institute. The second cluster is led by the University of Texas MD Anderson Cancer Center, primarily maintaining research relationships within the United States. The third and fourth clusters feature European institutions, notably led by Leiden University and Maastricht University, which collaborate closely, possibly due to the smaller patient populations in Europe necessitating multi-institutional research efforts. The fifth and sixth clusters represent research institutions in South Korea and Japan, spearheaded by Yonsei University and the University of Tokyo, respectively.

### 4.3 Journals and citations

The number of publications by a journal reflects its interest in the field. Summarizing the top 10 journals by publication volume, we find significant interest from oncology and cancer-specific journals. Analyzing the top 10 journals by citation count, we identify Annals of Oncology, Journal of Clinical Oncology (JCO), and The Lancet Oncology as leading publications in this field, particularly The Lancet Oncology, which predominantly features recent research. Submitting high-quality papers to these journals could be considered. Impact Factor (IF) serves as a metric for assessing the academic influence of publications. Among the top 10 journals by publication volume, the impact factors range from 3.2 to 4.9, with three journals among the top five having citation counts exceeding 40. This underscores that high-quality research in this field receives recognition from top journals, though overall research quality remains somewhat modest, highlighting the need for more high-caliber studies.

### 4.4 Keyword analysis

Keyword analysis helps identify focal points and research trends. Aside from “rectal cancer” and “chemoradiotherapy,” the most frequent keywords include “neoadjuvant chemoradiotherapy,” “prognosis,” “total mesorectal excision (TME),” “local recurrence,” “short-course radiotherapy,” and “clinical research.” These keywords reflect a focus on neoadjuvant chemoradiotherapy, total mesorectal excision for rectal cancer, short-course radiotherapy, local recurrence, long-term prognosis, and clinical research. The timeline view indicates that research before 2016 primarily focused on chemotherapy drug selection and local recurrence rates. From 2017 to 2019, there was a shift toward topics concerning surgery, short-course radiotherapy, and long-term outcomes. Post-2020, attention has predominantly been on multicenter clinical studies of locally advanced rectal cancer, total neoadjuvant treatment models, and immunotherapy.

### 4.5 Analysis of highly cited articles and future trends

Analyzing the top 10 most cited articles, it is evident that each year from 2014 to 2022 saw approximately one publication, with exceptions of three publications in 2016 and none in 2018. These 10 articles were sourced from three journals: “The Lancet Oncology” contributed three articles, “JCO” contributed three articles, and “Annals of Oncology” contributed two articles. In terms of thematic focus, three articles each explored the efficacy of radiotherapy combined with oxaliplatin and short-course radiotherapy. Additionally, one article investigated the total neoadjuvant therapy (TNT) model, another focused on sphincter preservation strategies, one examined the interval between radiotherapy and surgery, and another investigated the effects of triple-drug induction chemotherapy.

Studies on oxaliplatin efficacy were concentrated around 2015, while research on short-course radiotherapy emerged around 2017. In recent years, there has been significant exploration of the TNT model and anal sphincter preservation strategies. The primary objective of nine out of the 10 articles was to enhance both short-term and long-term efficacy in locally advanced rectal cancer, with one article focused on improving anal sphincter muscle function. This underscores efficacy and functional outcomes as central research directions. All articles were based on multicenter large-sample prospective randomized controlled trials.

Regarding study findings, introducing oxaliplatin during radiotherapy at 50 mg/m^2^ qw did not improve rates of pathological complete response (pCR) in the short term, but did enhance 3-year disease-free survival (DFS). Conversely, concurrent use of mFOLFOX during radiotherapy improved short-term outcomes, such as pCRs. Studies on short-course radiotherapy suggested that subsequent chemotherapy cycles post short-course radiotherapy achieves similar short- and long-term efficacy as conventional long-course chemoradiotherapy. Delayed surgery after short-course radiotherapy also contributes to reduced surgical complications. Induction chemotherapy with a triple-drug regimen before radiotherapy and the TNT model both demonstrated improvements in 3-year DFS, with TNT consolidative chemotherapy post-radiotherapy aiding in preserving anal sphincter function.

The potential role of neoadjuvant radiation dose intensifcation in locally advanced rectal cancer (LARC) has been a hot topic of research in recent years. Studies have shown that escalating the radiation dose to 60Gy in 30 fractions can result in better downstaging effects for patients with T3 rectal cancer. Although there is no significant difference in the pCR rate, the rate of sphincter-preserving surgeries has increased, and there is no significant statistical difference in the incidence of postoperative complications. However, acute genitourinary toxicities have increased, including cystitis, pelvic pain, urinary urgency, incontinence, and so on (32, 33). A more precise approach to selecting target lesions for radiation dose intensification may be a direction for future research.

Postoperative complications prevention and treatment are also hot topics in the medical field. Among these, Surgical Site Infection (SSI) is the most common postoperative complication after colorectal surgery, and it may even lead to sepsis, causing significant harm to patients. Recent literature has shown that anastomotic leakage is the most frequent cause of postoperative sepsis in colorectal surgery patients (34). Furthermore, radiotherapy is a high-risk factor for anastomotic leakage. Therefore, how to prevent postoperative SSI in patients who have undergone rectal radiotherapy and chemotherapy is a topic worth exploring. Recent studies have also indicated that Butyrylcholinesterase levels can be used to predict the occurrence of postoperative SSIs. Low levels of Butyrylcholinesterase on the first and third postoperative days were associated with an increased risk of developing SSIs (35).

The application of Artificial Intelligence (AI) in medicine is becoming a growing trend. Deep Learning (DL), based on AI technology, is being applied to the pathological diagnosis, endoscopic diagnosis, and CT diagnosis of colorectal cancer, potentially improving accuracy and effectiveness (36, 37). In the future, advancements in this technology could assist pathologists and endoscopists, providing them with an additional “eye” to detect more diagnostic details and reduce misdiagnosis and missed diagnoses.

Since 2014, laboratory research has indicated that radiotherapy can alter the immune microenvironment of colorectal cancer by activating antigen-presenting cells, recruiting T lymphocyte infiltration, and enhancing the efficacy of PD-1 inhibitors (38–40). In 2021, results from the NRS Gl-001 study were published in “JAMA Oncol.” This study enrolled locally advanced rectal cancer patients who underwent 4 months of FOLFOX induction chemotherapy followed by long-course radiotherapy concurrently with pembrolizumab. The results indicated a potential reduction in NAR scores in the study group, although without statistical significance. Updates at the 2023 ASCO-GI conference on long-term survival outcomes showed the study group achieved a significant improvement in 3-year overall survival (*p* < 0.05).

In 2024, “Annals of Oncology” published findings from the UNION study conducted in China (10). Similarly enrolling patients with locally advanced rectal cancer, the study randomized patients to receive either short-course radiotherapy plus two cycles of immunotherapy (PD-1 inhibitor + CAPOX) or long-course radiotherapy plus two cycles of CAPOX chemotherapy. Results showed a pCR of 39.8% in the study group compared to 15.2% in the control group, indicating significant improvement in short-term efficacy with manageable adverse effects. Given these notable efficacy improvements, future research directions worth exploring include radiotherapy combined with immunotherapy, the TNT model, and strategies for anal sphincter preservation.

## 5 Conclusion

Research on radiotherapy for rectal cancer is steadily increasing annually, particularly with a notable surge beginning in 2023. Scholars and institutions from China, the United States, and Europe play crucial roles in this field, with Chinese researchers notably asserting influence since 2020. High-quality publications in this area are being accepted by top-tier journals, suggesting a promising future for further high-quality prospective multicenter studies. “Prognosis” and “total neoadjuvant therapy” emerge as frequent key terms in current research, highlighting the trend toward investigating patterns and outcomes of preoperative chemoradiotherapy for locally advanced rectal cancer.

## Data Availability

The original contributions presented in the study are included in the article/supplementary material, further inquiries can be directed to the corresponding author.
